# Evaluation of the prognostic value of paraoxonase 1 in the recurrence and metastasis of hepatocellular carcinoma and establishment of a liver-specific predictive model of survival

**DOI:** 10.1186/s12967-018-1707-0

**Published:** 2018-11-26

**Authors:** Zheng Yu, Qifeng Ou, Fan Chen, Jiong Bi, Wen Li, Jieyi Ma, Rongchang Wang, Xiaohui Huang

**Affiliations:** 1 Laboratory of Surgery, The First Affiliated Hospital, Sun Yat-Sen University, Guangzhou, 510080 China; 2 Department of Orthopedics Surgery, The First Affiliated Hospital, Sun Yat-Sen University, Guangzhou, 510080 China; 3 Department of Gastrointestinal Surgery, The First Affiliated Hospital, Guangzhou Medical University, Guangzhou, 510120 China

**Keywords:** Hepatocellular carcinoma, Paraoxonase 1, Recurrence, Metastasis, Overall survival, Nomogram

## Abstract

**Background:**

Hepatocellular carcinoma is a malignant tumor with a highly invasive and metastatic phenotype, and the detection of potential indicators associated with its recurrence and metastasis after surgical resection is critical for patient survival.

**Methods:**

Transcriptome data for large cohorts (n = 1432) from multicenter sources were comprehensively analyzed to explore such potential signatures. The prognostic value of the selected indicators was investigated and discussed, and a comparison with conventional clinicopathological features was performed. A survival predictive nomogram for 5-year survival was established with the selected indicator using the Cox proportional hazards regression. To validate the indicator at the protein level, we performed immunohistochemical staining with paraffin-embedded slides of hepatocellular carcinoma samples (n = 67 patients) from our hospital. Finally, a gene set enrichment analysis (GSEA) was performed to detect the underlying biological processes and internal mechanisms.

**Results:**

The liver-specific protein paraoxonase 1 (PON1) was found to be the most relevant indicator of tumor recurrence, invasiveness, and metastasis in the present study, and the downregulation of PON1 might reveal poor survival for patients with hepatocellular carcinoma. The C-index of the PON1-related nomogram was 0.714, thus indicating a more effective predictive performance than the 7th American Joint Committee on Cancer (AJCC) tumor stage (0.534), AJCC T stage (0.565), or alpha-fetoprotein (0.488). The GSEA revealed that PON1 was associated with several hepatocellular carcinoma-related pathways, including the cell cycle, DNA replication, gap junction and p53 downstream pathways.

**Conclusions:**

The downregulation of paraoxonase 1 may suggest worse outcomes and a higher recurrence rate. Thus, paraoxonase 1 might represent an indicator for predicting the survival of patients with hepatocellular carcinoma.

**Electronic supplementary material:**

The online version of this article (10.1186/s12967-018-1707-0) contains supplementary material, which is available to authorized users.

## Background

Recurrence and metastasis after hepatic resection of hepatocellular carcinoma (HCC) usually contributes to poor long-term patient survival [1]. HCC has strong invasiveness and metastasis abilities, thus enhancing its recurrence rate [2]. Although novel therapies have been developed in recent years, the mortality of HCC patients has not decreased [3]. The identification of potential indicators associated with recurrence and metastasis can improve treatment response in clinical trials and the quality of life of HCC patients. Transcriptome data from very large cohorts were used in the present study to show that paraoxonase 1 (PON1) might represent a potential signature that is strongly correlated with HCC recurrence and metastasis.

Evidence has shown that PON1 is a member of the paraoxonase family, and it encodes a protein of an enzyme with lactonase and ester hydrolase activity [4]. It is an antioxidant defensive factor that is relevant in the pathogenesis of several inflammatory diseases [5]. Increasing evidence has demonstrated that PON1 plays a significant role in atherosclerosis as its downregulation may lead to the disease [6, 7]. In addition, its clinical application value in cancer studies has been gradually discovered [8]. Increasing evidence has demonstrated that PON1 could serve as a significant clinical indicator for breast cancer and lung cancer [9, 10]. Meanwhile, several studies have also found that serum PON1 is highly fucosylated (Fuc-PON1) and could be utilized as a novel diagnostic biomarker of early-stage HCC [11]. However, the number of studies on PON1 in HCC is limited, and its latent prognostic value and potential in clinical applications, especially its correlation with metastasis, recurrence, overall survival (OS) and other clinical risk factors, has not been observed.

In the present study, we performed a bioinformatics analysis based on sequencing data of 1432 patients’ tissues to find reliable prognostic signatures. A weighted correlation network analysis (WGCNA) was utilized using The Cancer Genome Atlas (TCGA) for a blind selection in our research. Latent prognostic indicator was selected for further analysis. Expression difference of selected indicator in tumor tissue and nontumor tissue was validated in 7 Gene Expression Omnibus (GEO) datasets from multicenters. We also established a 5-year predictive model that was visualized with a nomogram for predicting 5-year survival. Subsequently, we validated our results on protein levels via experimental tools based on clinical data and our own samples. Finally, we detected and discussed related biological pathways and how they are affected in HCC with PON1 downregulation.

## Materials and methods

### Transcriptome data of HCC patients in the present study

Data retrieved from multiple research centers were used for integrated analysis in this study, including data from The Cancer Genome Atlas (TCGA) project and Gene Expression Omnibus (GEO) databases. We systematically analyzed the expression profiles of transcriptomes from the following datasets to ensure the credibility of the current study: TCGA [n = 423, National Cancer Institute (NCI) and the National Human Genome Research Institute (NHGRI), USA], GSE14323 (n = 124, Virginia Commonwealth University), GSE14520 (n = 481, National Cancer Institute, Laboratory of Human Carcinogenesis, USA), GSE6764 (n = 75, Mount Sinai School of Medicine), GSE51401 (n = 64, Zhongshan Hospital affiliated with Fudan University), GSE41804 (n = 40, Kanazawa University Graduate School of Medical Sciences), GSE45436 (n = 134, National Yang-Ming University) and GSE62232 (n = 91, INSERM, UMR U-1162, Université Paris Descartes). The sequencing data used in the present study were collected on Affymetrix microarray and Illumina platforms by different researchers. Complete data on the clinicopathologic characteristics of HCC patients in TCGA were obtained from Cbioportal (http://www.cbioportal.org/), including 7th American Joint Committee on Cancer (AJCC) staging rules, serum alpha-fetoprotein (AFP) level, disease-free time, OS time, living status and recurrence time.

### Identification of differentially expressed genes

Increasing evidence has demonstrated that the expression levels of cancer-related genes are abnormally changed during the initiation of HCC. Thus, discovering these genes for further analysis is critical. Based on 373 tumor tissues and 50 adjacent nontumor tissues in TCGA datasets, we identified key differentially expressed genes (DEGs) via R/language (edgeR package, R version 3.34). Varying degrees of gene expression between tumor tissues and adjacent non-tumor tissues were evaluated by *log2*(*fold*-*change*). Genes with abs(*log2*(*fold*-*change*)) > 1 (absolute value) and P < 0.05 were selected as candidate signatures for further analysis.

### Weighted correlation network analysis for discovering recurrence-related gene modules

To uncover the recurrence-related DEGs, we conducted a WGCNA using DEGs [12, 13]. In general, genes with similar expression patterns are likely to exhibit coexpressed relationships and similar molecular functions. According to the connectivity of the coexpressed genes, WGCNA clustered thousands of genes into several gene modules using a soft thresholding power [14, 15]. The relations between different gene modules and clinicopathologic characteristics of prognosis were revealed by Pearson correlation. In the present study, we investigated gene modules that were the most relevant to recurrence and metastasis. We performed the WGCNA analysis based on clinical data and sequencing data of 373 HCC samples from TCGA dataset. Samples with the duplicated TCGA barcodes and uncomplete survival data were excluded. In total, 368 HCC patients were involved in the WGCNA, and the clinical information of these patients was also available for the WGCNA. The soft thresholding power was identified using the WGCNA algorithm and sequencing data of DEGs. Then, DEGs were clustered in different gene modules using the soft thresholding power, and the recurrence-related gene module was investigated.

### Indicator selection procedure

The Kaplan–Meier method was performed for every gene in the selected gene module from the WGCNA. The gene with the most significantly different log-rank p value was retained as our final selected indicator, and its expression difference between tumors and nontumors was validated using the GEO datasets. The prognostic value of our candidate indicator was also investigated. The relationship between the clinicopathologic characteristics and expression of the candidate indicator was also investigated. Student’s *t*-test was utilized for continuous variables, and Fisher’s exact test was used for categorical variables. In total, 369 patients with complete clinicopathologic information were evaluated. Density plots were illustrated for clear views.

### Prognostic nomogram with selected indicator

Patients with HCC usually have a poor prognosis due to tumor recurrence. Thus, establishing a recurrence-related model for predicting survival is significant. We established a nomogram to predict 5-year survival. Multivariate Cox proportional hazards regression was used for the nomogram. TCGA datasets of 368 HCC patients were used [16]. Compared with the traditional method, PON1 expression (log2 transformed, Htseq-counts) was used as a novel variable for the establishment of a nomogram. For other variables, we selected several survival-related indicators and basic information, including age, sex, AJCC staging indicators, tumor differentiation, vascular invasion, and Child–Pugh classification. Five-year survival prediction performance was examined with the C-index. To avoid the potential bias caused by tumor heterogeneity, we performed internal validation by extracting 60% of samples randomly as the validation dataset for 10 times. C-index was calculated for testing the robustness. Calibration curves were also illustrated to test the predicting accuracy. Univariate and multivariate COX proportional hazards tests were performed for conventional clinical features, and the receiver operating characteristic (ROC) curves of conventional features for predicting 5-year survival were also displayed for comparison.

### Immunohistochemical staining for validation at protein level

To validate the selected indicator at protein level, we performed immunohistochemical staining in the samples of human tissues. The slides of paraffin-embedded HCC tissues from 67 patients were obtained after surgical resection. All patients received radical resection without preoperative chemotherapy or radiotherapy in the First Affiliate Hospital of Sun-yat Sen University. All diagnoses were confirmed by pathology. The slides were incubated for 2 h at 65 °C, deparaffinized, and rehydrated. Retrieval of the heat-mediated antigen was conducted in 10 mmol/L Tris-citrate buffer (pH 7.0) with a pressure cooker. Blocking of endogenous peroxidase activity was performed by incubating sections with 3% hydrogen peroxide for 10 min at room temperature. Each section was then incubated with 5% normal goat serum in phosphate-buffered saline containing 0.1% Tween 20 for 30 min at room temperature to block nonspecific binding of the primary antibody. The slides were incubated with primary antibodies (diluted 1:250) against PON1 (Abcam, ab92466) overnight at 4 °C. After washing, each slide was incubated with the appropriate horseradish peroxidase (HRP)-labeled secondary antibody and then developed with DAB solution (DAKO, Agilent) before counterstaining with hematoxylin. Staining intensity was scored as 0, 1, 2, or 3 for negative, weak, moderate, or strong, respectively, and the staining percentage was given a score of 0 (absent) for < 5% positive staining, 1 (focal) for 5% to < 50% positive staining, or 2 (diffuse) for ≥ 50% positive staining. The sum of the intensity and distribution scores was then used to determine PON1 immunoreactivity. A score of 1 or 0 was considered to show low expression, whereas higher scores were considered to indicate high expression. Two pathologists assessed the specimens independently. Images were obtained using an Olympus BX63 microscope (Olympus, Japan).

### Biological pathway and internal mechanism detection

To investigate the pathways and biological parameters correlated with PON1, we conducted a gene set enrichment analysis (GSEA) [17, 18]. The 368 HCC patients in the TCGA dataset were separated into high-expression and low-expression groups according to the median value of PON1 and retained as the phenotypes. GSEA software was obtained from GSEA website (http://software.broadinstitute.org/gsea/index.jsp). One thousand permutations were performed to determine the statistically significant and ensure the credibility of the results. Pathways with a FDR < 0.05 and P-value < 0.05 were selected as the enriched terms. The molecular signatures database from the GSEA were utilized as the annotation file. Pathways from the Kyoto Encyclopedia of Genes and Genomes (KEGG) and Reactome and Pathway Interaction Database (PID) were used. A Gene Ontology enrichment analysis was also performed to discover biological functions in HCC. Correlated biological processes, molecular functions, and cellular components were identified.

### Statistical software and data format

DEGs were identified using edgeR (Bioconductor/R version 3.34) [19, 20]. Htseq-counts (TCGA) and RSEM (GEO, log2 scaled) were used as the sequencing data formats. WGCNA was performed by the WGCNA package (R version 3.3.4). Nomogram was established using Regression Modeling Strategies (RMS, R version 3.34). Student’s *t*-test and Fisher’s exact test were performed in R (version 3.34).

## Results

### Identification of differentially expressed genes

**Fig. 1:**
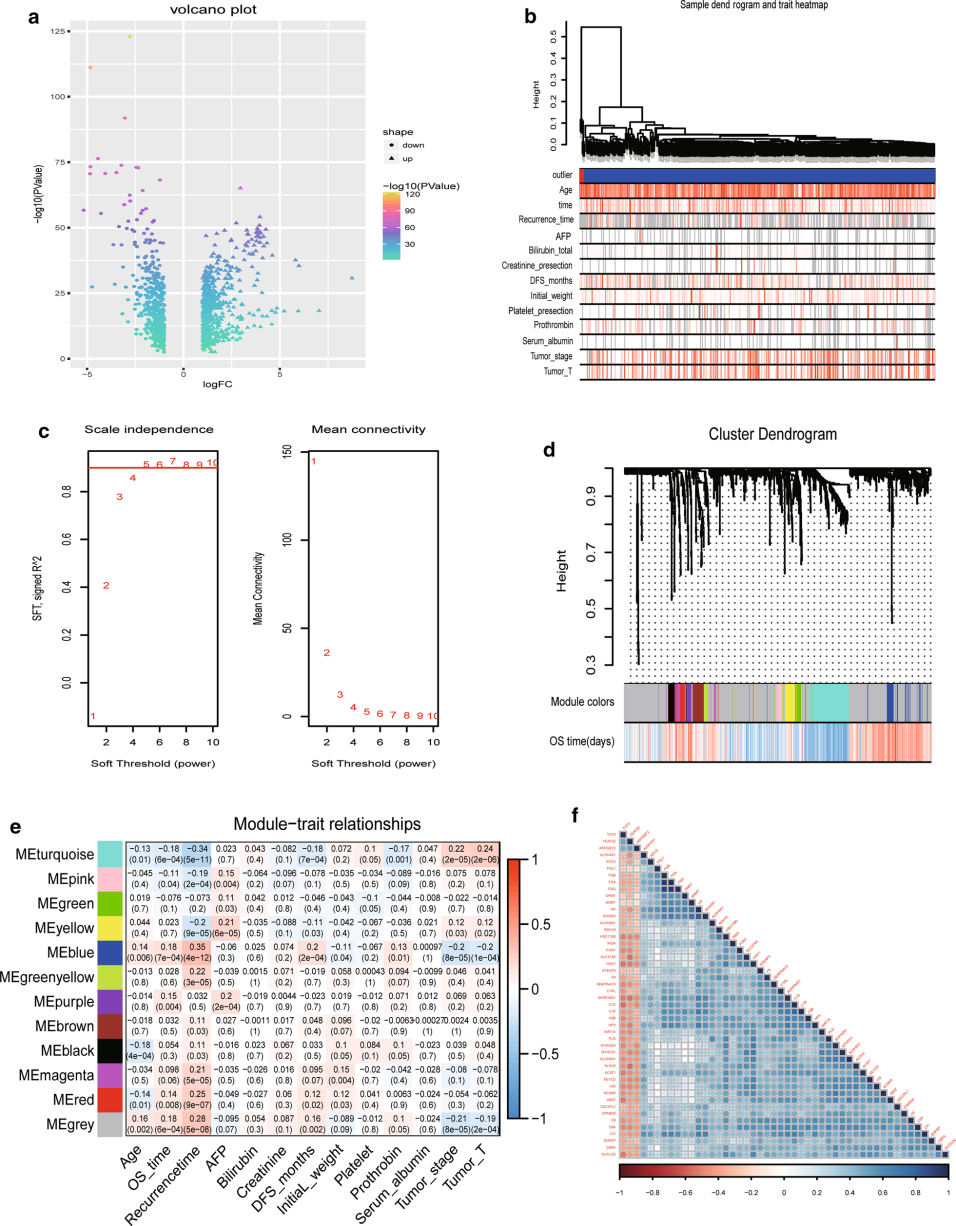
Identifying recurrence-related prognostic indicators. **a** Volcano plot exhibiting the distribution of DEGs via *log2*(*fold*-*change*) and P values. **b** Sample clusters showing basic clinical information on HCC patients. **c** Soft threshold power selection for gene clustering; a value of 7 was selected as the threshold. **d** In total, 13 gene modules were discovered via gene clustering. **e** Heatmap exhibiting the relationship between different gene modules and clinical risk factors (Pearson correlation). Blue modules were the most relevant to recurrence and OS. **f** Plot showing 34 genes in blue modules and their coexpression relationships

A total of 985 DEGs were discovered by bioinformatics analysis, including 512 upregulated and 473 downregulated genes (Additional file 1: Table S1). The variation degree of DEGs ranged from 8 to − 5 as evaluated by *log2*(*fold*-*change*). A volcano plot was created to illustrate the distribution of DEGs (Fig. 1a).

### WGCNA analysis revealed blue gene module was related to tumor recurrence

Based on the complete clinical information and RNA-seq data of 985 DEGs (Htseq-count), we performed WGCNA analysis to cluster DEGs into different modules according to their coexpression relationships. We also revealed different gene modules and their relevant clinical information. Patients were first clustered to show basic clinical information (Fig. 1b). Then, a soft threshold power was calculated for gene clustering, and 7 was selected as the power in the present study (Fig. 1c). In total, 13 gene modules were discovered (Fig. 1d). The relationships between gene modules and primary clinical indicators were discovered with Pearson correlation. Correlation coefficients (*r*) were illustrated as a heatmap (Fig. 1e). Blue modules were positively correlated with OS time (*r* = 0.18) and recurrence time (*r* = 0.35). In addition, they were also negatively correlated with AJCC tumor staging rules, including T stage (*r* = − 0.20) and tumor stage (*r* = − 0.20). The results demonstrated that genes in the blue modules could be viewed as tumor suppressors. Patients with higher expression of genes in blue modules had longer survival times. A total of 34 genes were found in blue modules (Fig. 1f). Survival analysis was performed with genes in blue module. PON1 was the most relevant gene for OS using the Kaplan–Meier (KM) method according to log-rank P values (Additional file 2: Table S2).

### Reduction in PON1 expression indicated greater invasiveness and a poor prognosis

**Fig. 2:**
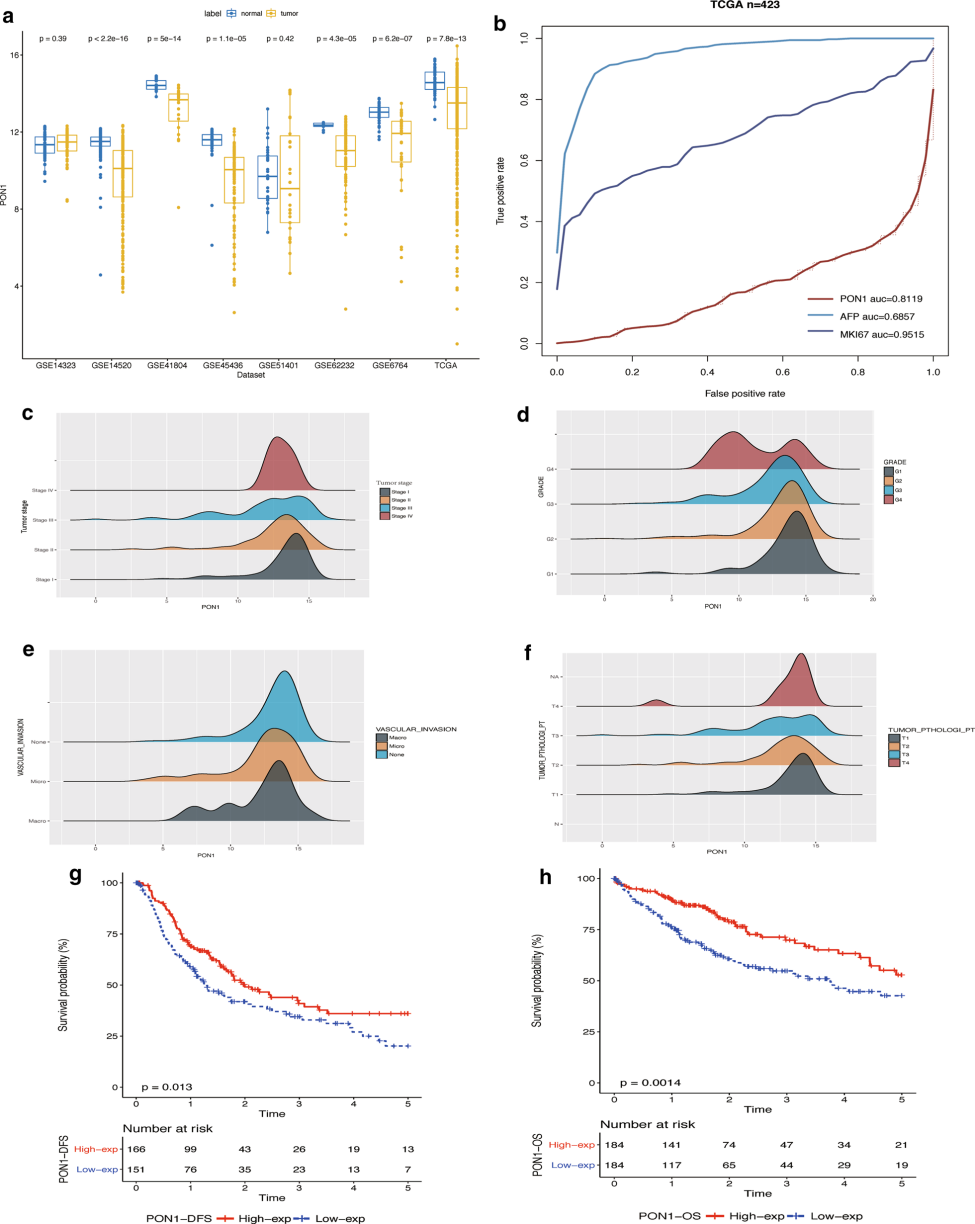
Prognostic value of PON1 in clinical applications. **a** Validation of PON1 expression differences in tumor tissues and adjacent non-tumor tissues by analyzing multicenter data sources that included 1432 samples. PON1 was downregulated in HCC tissues. **b** Comparison of the diagnostic ability of PON1, MKI67, and AFP via ROC curves. The diagnostic accuracy of PON1 (0.8119) was higher than that of AFP (0.6857) and lower than that of MKI67 (0.9515). **c**–**f** Density plot exhibiting the relationship between PON1 expression and clinicopathologic characteristics, including AJCC tumor stage, tumor T stage, tumor differentiation, and vascular invasion. The trend in the peaks indicated that low tumor expression was likely to be classified as a poor prognosis, including stage III, G4, macro vascular invasion, and T4. **g** Disease-free survival analysis of patients with low PON1 expression and high PON1 expression in the TCGA dataset (P-value = 0.013). **h** OS analysis of patients with low PON1 expression and high PON1 expression in TCGA dataset (P = 0.0014)

We detected the expression of PON1 in other GEO datasets and found that PON1 expression in HCC was downregulated in nearly all datasets (Fig. 2a, Additional file 3: Table S3). Patients in the TCGA dataset were separated into two groups according the median value of PON1, including a high-expression group and a low-expression group. Differences between the two groups were investigated with Student’s *t*-test and Fisher’s exact test (Table 1). The results revealed that significant differences existed in age, sex, AJCC TNM staging rules, tumor differentiation, and vascular invasion. We compared the diagnosis performance of key clinical indicators in HCC, including marker Ki-67 (MKI67), AFP, and PON1 [21, 22]. The results indicated that the area under curve (AUC) of PON1 was 0.8119, which was higher than that of AFP (0.6857) but lower than that of MKI67 (0.9515) (Fig. 2b) [23]. Based on a density plot of PON1 expression, we clearly observed that low PON1 expression in HCC was correlated with stronger invasiveness and metastasis, including tumor T stage, AJCC tumor stage, tumor differentiation and vascular invasion (Fig. 2c–f) [24]. The KM curves of Disease-free survival and overall survival was present for visualizing survival data, whilst the Log Rank test was utilized for determining differences. Results revealed Log Rank P-values were 0.013 (DFS) and P = 0.0014 (OS) separately (Fig. 2g, h). HCC patients with low PON1 expression were found to have a poor prognosis in long-term survival.

**Table 1:** Association of PON1 with clinicopathological characteristics of HCC

Clinical factors	PON1 level		Total (n = 369)	95% CI		P-value
Group	High (n = 184)	Low (n = 185)				
Gender						
F	40	81	121	0.2198819	0.575337	8.10E−06
M	144	104	248			
Age (mean, SD)	61.17	57.64		0.7774031	6.287814	0.01212
Tumor pathologic_stage						
Stage I–II	145	129	256	1.140819	3.267362	0.009873
Stage III–IV	36	56	89			
Tumor size						
T1–T2	145	129	274	1.052772	2.921888	0.02976
T3–T4	36	56	92			
Tumor metastasis						
M0	129	136	265	0.2244094	150.4063	0.623
M1	1	3	4			
Tumor nodes						
N0	120	130	250	0.2181723	146.4568	0.6237
N1	1	3	4			
Child–Pugh classification						
A	115	101	216			0.1671
B	15	6	21			
C	1	0	1			
Grade						
G1	36	19	55			9.62E−05
G2	99	77	176			
G3	42	79	121			
G4	4	8	12			
Race						
American indian or alaska tive	1	1	2			0.1536
Asian	68	90	158			
Black or african american	10	7	17			
White	98	84	182			
Tumor status						
Tumor free	119	114	233	0.7075	1.8518	0.6426
With tumor	52	57	109			
Vascular invasion						
Macro	6	9	15			0.00552
Micro	35	57	92			
Non-vascular invasion	118	88	206			
Hepatic_infalmmation_adj_tissue						
Mild	44	55	99			0.02331
Severe	10	8	18			
None	73	43	116			
AFP (mean, SD)	16352.55	11481.7				0.7408

### PON1-related prognostic nomogram

**Fig. 3:**
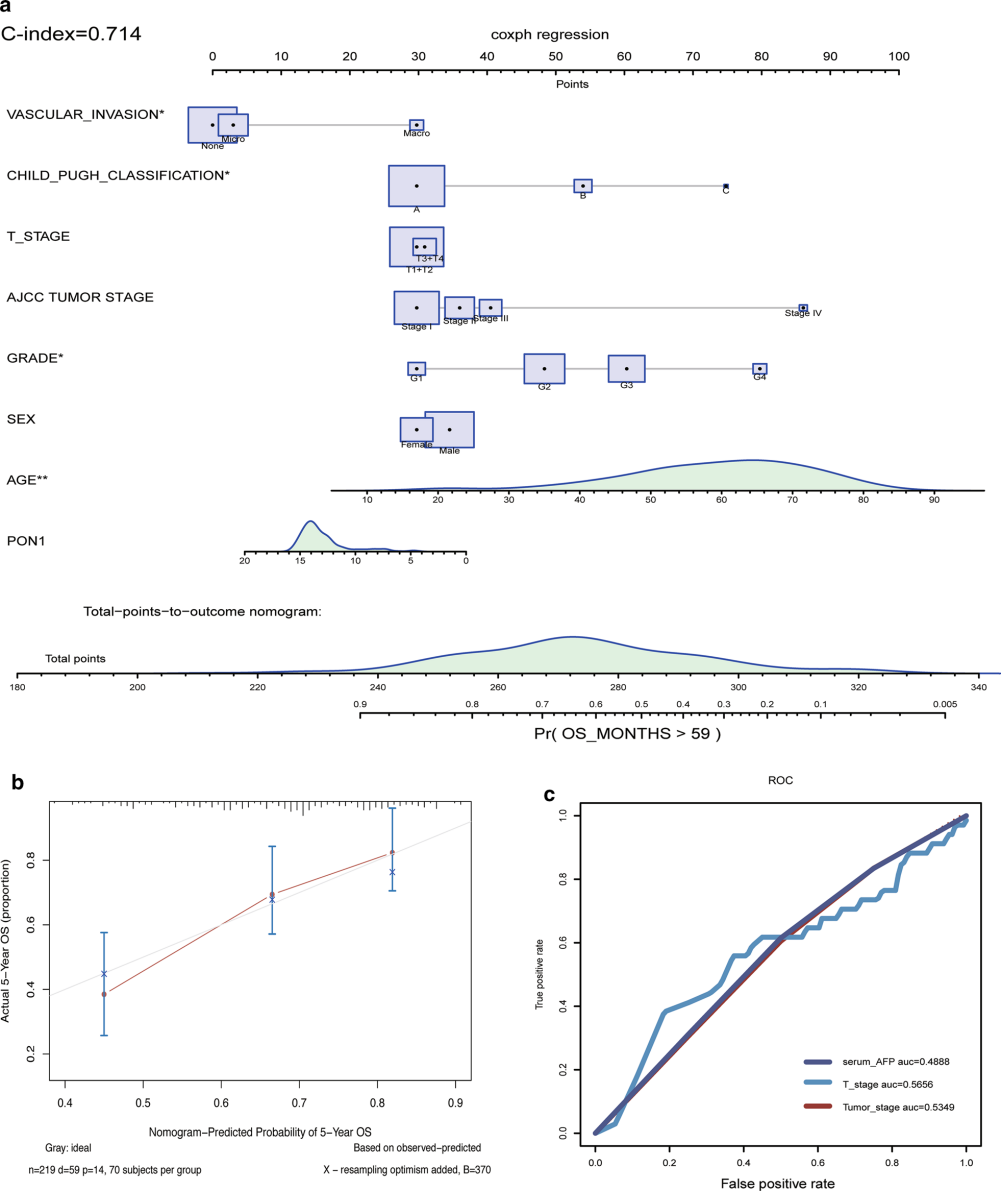
Establishment of the prognostic nomogram for 5-year survival. **a** Nomogram for predicting survival probabilities. Each clinical characteristic is shown with its corresponding score. PON1 was used as a novel liver-specific variable in the nomogram. The C-index of the nomogram was 0.714. **b** Calibration plot of OS at 5 years to visualize the difference between true values and predicted values. **c** ROC curves of AJCC tumor stage (0.5349), serum AFP (0.4888), and tumor T stage (0.5656) for predicting 5-year survival are shown for comparison

A prognostic nomogram was established between PON1 and several significant clinical factors, including age, sex, vascular invasion, Child–Pugh classification, AJCC staging rules, and tumor differentiation (Fig. 3a). The calibration curves indicated that the predictive performance of the model was excellent (Fig. 3b). In our nomogram, all variables were in accordance with clinical logic. PON1 was utilized as a new variable and promoted model accuracy. The expression of PON1 was negatively correlated with risk score. The C-index (an evaluating marker similar to the ROC curve) of the model was 0.714 (0.6753–0.7627, C-index in validation datasets). The results of the univariate Cox hazards analysis revealed that the AJCC tumor pathological stage, tumor size, tumor metastasis, and AFP level were related to 5-year survival. Hazard ratios were listed (Additional file 4: Table S4). As a comparison with the nomogram, we illustrated the ROC curves using the AJCC tumor stage, tumor size, and AFP in 5-year survival prediction (Fig. 3c). The results indicated that our nomogram provided a more accurate prediction than using conventional clinical features.

### Experimental validation of PON1 at the protein level by immunohistochemistry

**Fig. 4:**
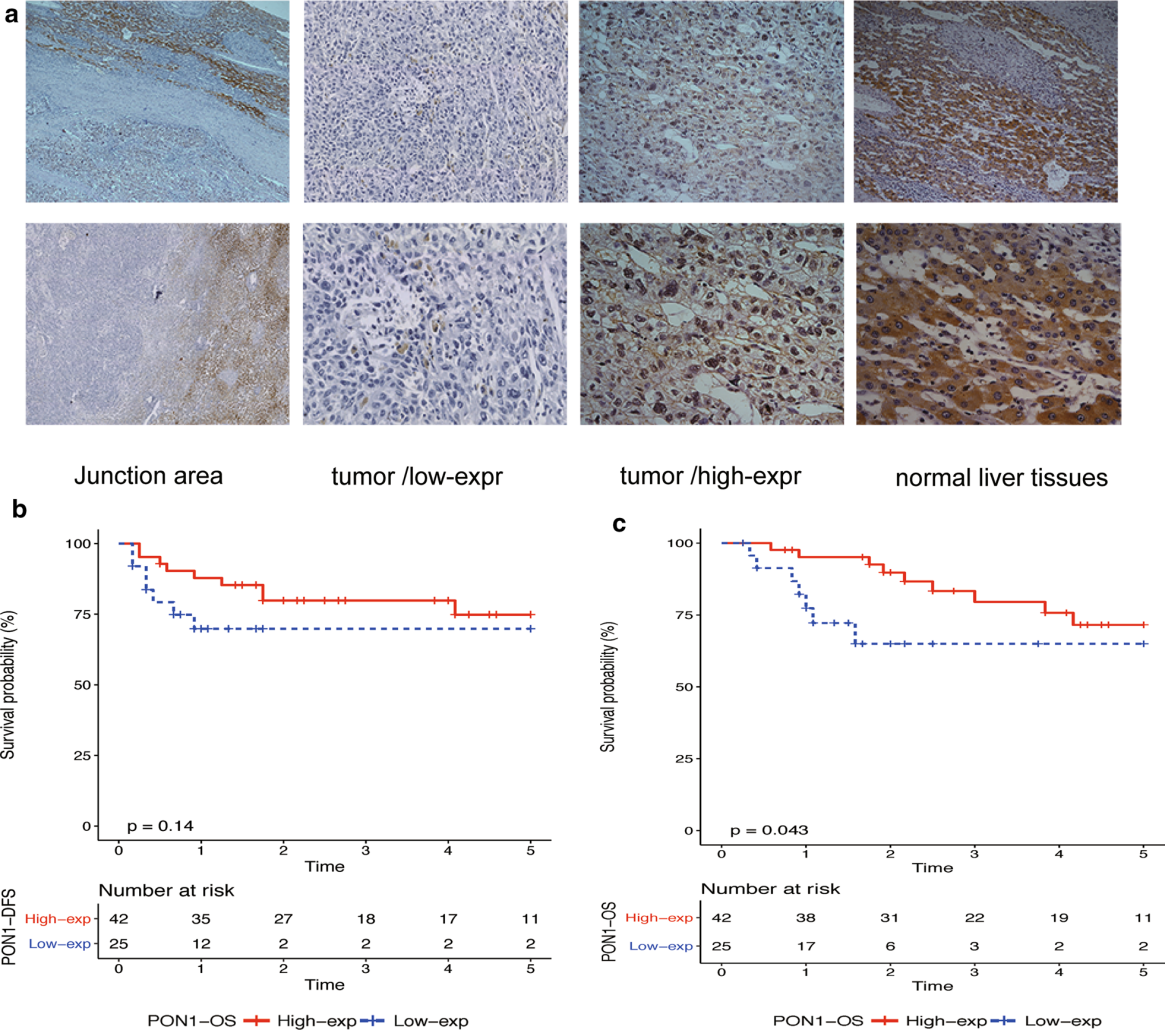
Protein level validation using immunohistochemistry (IHC). PON1 protein expression in adjacent non-tumor tissues and tumor tissues. **a** PON1 location in HCC was demonstrated, particularly in the cytoplasm. Low and high expression in tumor tissues was also illustrated. The junction area is shown as a comparison. **b** Disease-free survival based on the IHC of 67 patients using the KM method (P = 0.14). **c** OS based on the IHC of 67 patients using the KM method (P = 0.043)

To validate these results, we conducted an immunohistochemical analysis to reveal the protein level of PON1 in 67 HCC patients to explore the relationship between PON1 and HCC clinicopathological characteristics. PON1 was found to be significantly located in the cytoplasm, and it was downregulated in HCC tissues. The junction area was illustrated for the comparison. The statistical results indicated that 42 (62.7%) of the 67 samples showed high expression levels according to staining intensity (Fig. 4a). Survival data were also available for 67 patients. KM curves of the OS and disease-free survival was presented for visualizing survival data, whilst the Log Rank test was performed for determining differences. Result revealed that patients with low expression of PON1 in HCC tissue had a poor prognosis for overall survival (Fig. 4b, c).

### Correlated biological pathways of PON1 in HCC

**Fig. 5:**
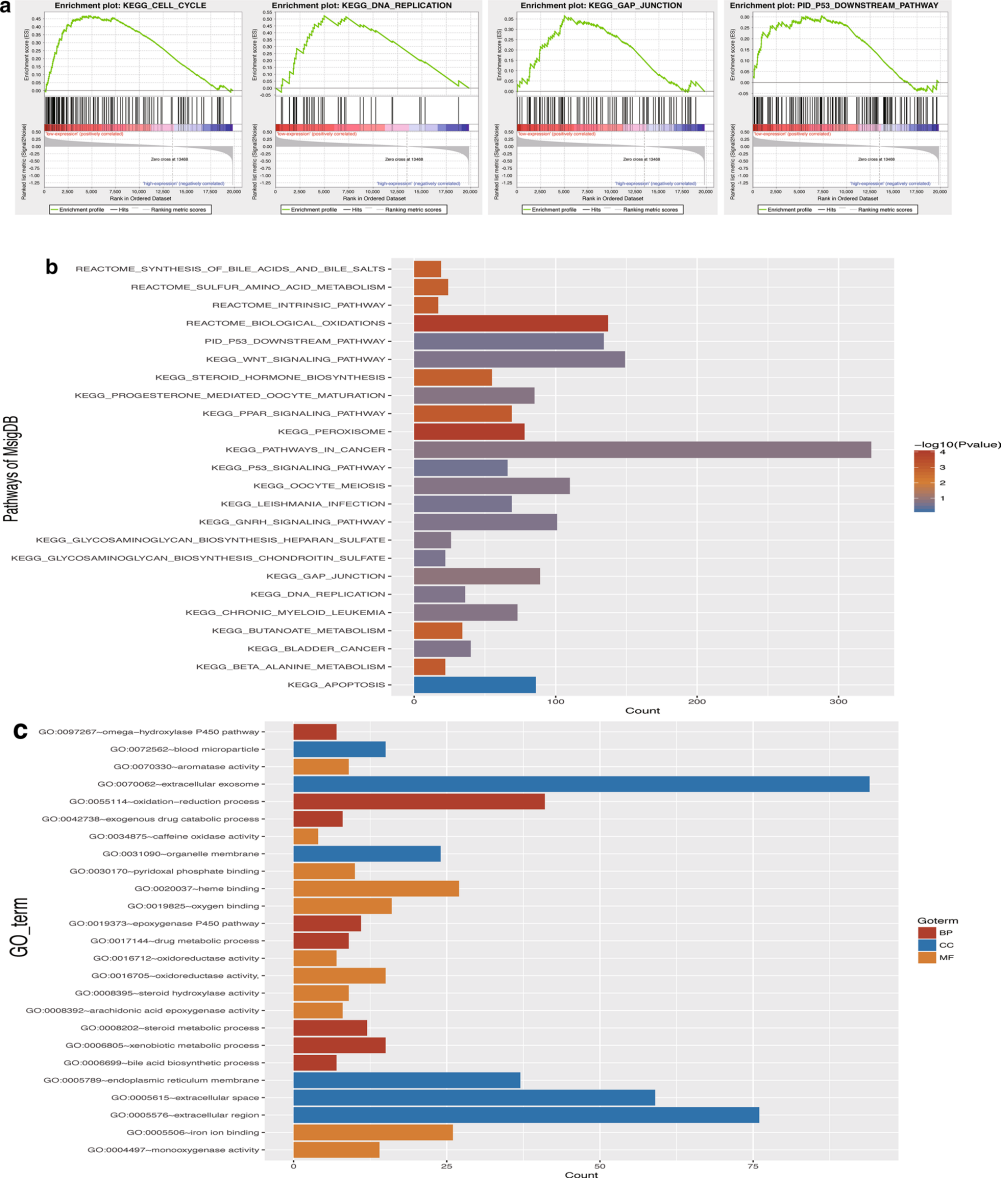
Detection of biological pathways and internal mechanisms. **a** Plot of the GSEA; several key pathways are visualized, including the cell cycle, DNA replication, gap junction, and p53 downstream pathways. **b** Enriched pathways found by the GSEA using MsigDB. **c** Plot of the Gene Ontology enrichment analysis. Biological processes (BP), molecular functions (MF) and cellular components (CC) are illustrated

The GSEA was used to detect pathways that were correlated with PON1. HCC-related pathways were found to be related to PON1, including the cell cycle (P = 0.00122), DNA replication (P = 0.002093), gap junction (P = 0.01286), and p53 downstream pathways (P = 0.00252) (Fig. 5a, Additional file 5: Table S5). Researchers have found that the cell cycle pathway is abnormally changed from normal liver functions to chronic hepatitis as well as during the transition into HCC [25]. Increasing evidence has revealed that gap junction pathways could affect HCC invasion and metastasis [26]. Apoptosis was also discovered to be related to PON1 (P = 0.008673). As a reference, peroxisome (P = 0.0001) and biological oxidation pathways (P = 0.0001) were also identified (Fig. 5b, c) [27]. These findings demonstrated that the results of the GSEA were reliable. Enrichment of Gene Ontology terms was also conducted and illustrated. The results indicated that PON1 variation in HCC leads to changes in oxidation reduction processes (P < 0.001), oxygen binding (P < 0.001), extracellular exosomes (P < 0.001), and blood microparticles (P < 0.001) (Additional file 6: Table S6).

## Discussion

Although many HCC-related prognostic biomarkers have been identified in recent years, most do not exhibit tissue specificity [28]. Thus, these biomarkers might be affected by various factors, and they lack significant value in clinical applications. We performed a systematic analysis that discovered the prognostic value of PON1 in patients with HCC. We found that PON1 downregulation in HCC suggests worse tumor differentiation, higher recurrence rate, stronger invasiveness, and poorer outcomes. Researchers in recent years have demonstrated that serum PON1 levels can assist in diagnosing AFP-negative HCC at an early stage [29, 30]. Moreover, PON1 played an important role in the initiation of non-alcoholic fatty liver disease (NAFLD) [31, 32]. Meanwhile, NAFLD was found to be a potential risk factor for HCC, especially in patients without hepatitis virus infection [33, 34]. However, discoveries about PON1 in HCC were mostly based on small cohorts from a single data source in retrospective studies, which ignored diversity in terms of race, age, and hepatitis virus infection. The prognostic application of PON1 was also rarely investigated or discussed. Finding crucial cancer biomarkers from thousands of genes is usually difficult. In the present study, we introduced WGCNA algorithm for discovering recurrence-related indicators. Compared with other algorithms, WGCNA systematically combined the sequencing data and clinical information. Using of WGCNA could assist in discovering underlying clinical significance of some key genes. WGCNA also illustrated the connection of genes with similar expression patterns. Moreover, WGCNA algorithm was gradually used in analyzing the single-cell transcriptome data by researchers [35]. Meanwhile, our research was conducted on 1432 samples from multicenter data sources, and the prognostic and predictive values were validated at both the gene expression and protein level. We also detected possible internal mechanisms and biological processes related to HCC. Therefore, we promoted other researchers’ studies and provided more credible results.

Even though the expression of PON1 is negatively correlated with tumor recurrence, metastasis, and invasion, we did not define it as a tumor suppressor. The PON1 gene encodes an enzyme that can be released from normal liver cells into the blood circulation and is of great antioxidant significance [36, 37]. According to our pathway detection results, PON1 downregulation may not directly affect the invasiveness and metastasis of tumor cells as PON1 is located downstream of cancer-related biological processes. In our view, PON1 downregulation in HCC might be induced by losing the original function of normal liver cells in PON1 secretion. In GSEA analysis, the results indicated that several carcinogenesis-related pathways were enriched by varying degrees by PON1, including the p53 downstream pathway, gap junction, cell cycle, apoptosis, and DNA replication. Therefore, PON1 downregulation might be increased by complicated internal mechanisms related to cell division, proliferation, and migration, which could also explain why the expression of PON1 was related to tumor recurrence and clinical outcomes. However, a decrease in PON1 might still cause changes in tumor cells, especially tumor-derived inflammation, autophagy, and apoptosis [38, 39]. However, we still need more experimental evidence to prove our conclusions from sequencing data and pathological results. In addition, further detection of these mechanisms will be very necessary.

In recent years, next-generation sequencing (NGS) use has gradually increased [40, 41]. However, NGS in tumors is still based on tissue obtained from surgical resection, which limits the clinical application of this method. Although some novel test methods, including sequencing of tumor-derived circulating DNA and exosomes in the blood, have been discovered, their practicability still needs to be validated [42–44]. Noninvasive methods, including blood tests, are still very important. Published research has shown that PON1 is an enzyme that is mainly synthesized in the liver and released into the circulatory system [45, 46]. The expression of PON1 is mainly affected by liver cells, and the use of PON1 in a predictive model may reduce other possible interference factors. Therefore, we built a PON1-related nomogram with a Cox proportional hazards regression to investigate 5-year patient survival. In our model, PON1 gene expression was quantified and served as an indicator to promote its specificity. Compared with conventional clinical indicators, our nomogram exhibited excellent prediction accuracy and effectiveness. However, the limitation of our model is that we did not examine serum PON1 levels or use them as a variable. The model will be more useful if serum PON1 levels are tested and used as a variable.

## Conclusions

In summary, we performed a comprehensive analysis of the prognostic value of PON1. We discovered that PON1 downregulation indicates a high recurrence rate and poor outcomes. We also provided a nomogram to use PON1 in clinical applications. We supplied a more accurate plan than conventional methods for predicting prognosis.

## Additional files

**Additional file 1.** 985 differentially expressed genes in HCC.

**Additional file 2.** Log-rank P values of genes in blue module.

**Additional file 3.** Expression values of PON1 in 8 HCC datasets from TCGA and GEO databases.

**Additional file 4.** Univariate and multivariate Cox proportional-hazard regression analysis for overall survivalin TCGA dataset (HCC).

**Additional file 5.** GSEA for MSigDB pathways (KEGG, PID, REACTOME)

**Additional file 6.** Gene ontology (GO) enrichment analysis.

## Abbreviations

PON1: paraoxonase 1
HCC: hepatocellular carcinoma
TNM: tumor node-metastasis
WGCNA: weighted correlation network analysis
GSEA: gene set enrichment analysis

## References

[1] Sparchez Z, Mocan T (2017). Hepatocellular carcinoma occurrence and recurrence after antiviral treatment in HCV-related cirrhosis. Are outcomes different after direct antiviral agents? A review. J Gastrointestin Liver Dis.

[2] Maluccio M, Covey A (2012). Recent progress in understanding, diagnosing, and treating hepatocellular carcinoma. CA Cancer J Clin.

[3] Llovet JM, Burroughs A, Bruix J (2003). Hepatocellular carcinoma. Lancet.

[4] Mackness M, Mackness B (2015). Human paraoxonase-1 (PON1): gene structure and expression, promiscuous activities and multiple physiological roles. Gene.

[5] Borovkova EI, Antipova NV, Komeenko TV, Shakhparonov MI, Borovkov IM (2017). Paraoxonase: the universal factor of antioxidant defense in human body. Vestn Ross Akad Med Nauk.

[6] Moya C, Manez S (2018). Paraoxonases: metabolic role and pharmacological projection. Naunyn Schmiedebergs Arch Pharmacol.

[7] Shih DM, Gu L, Xia YR, Navab M, Li WF, Hama S, Castellani LW, Furlong CE, Costa LG, Fogelman AM, Lusis AJ (1998). Mice lacking serum paraoxonase are susceptible to organophosphate toxicity and atherosclerosis. Nature.

[8] Bacchetti T, Ferretti G, Sahebkar A (2017). The role of paraoxonase in cancer. Semin Cancer Biol.

[9] Bobin-Dubigeon C, Lefrancois A, Classe JM, Joalland MP, Bard JM (2015). Paired measurement of serum amyloid A (SAA) and paraoxonase 1 (PON1) as useful markers in breast cancer recurrence. Clin Biochem.

[10] Aldonza MBD, Son YS, Sung HJ, Ahn JM, Choi YJ, Kim YI, Cho S, Cho JY (2017). Paraoxonase-1 (PON1) induces metastatic potential and apoptosis escape via its antioxidative function in lung cancer cells. Oncotarget.

[11] Zhang S, Jiang K, Zhang Q, Guo K, Liu Y (2015). Serum fucosylated paraoxonase 1 as a potential glycobiomarker for clinical diagnosis of early hepatocellular carcinoma using ELISA Index. Glycoconj J.

[12] Miller JA, Cai C, Langfelder P, Geschwind DH, Kurian SM, Salomon DR, Horvath S (2011). Strategies for aggregating gene expression data: the collapseRows R function. BMC Bioinform.

[13] Zhang B, Horvath S (2005). A general framework for weighted gene co-expression network analysis. Stat Appl Genet Mol Biol.

[14] Langfelder P, Luo R, Oldham MC, Horvath S (2011). Is my network module preserved and reproducible?. PLoS Comput Biol.

[15] Langfelder P, Horvath S (2008). WGCNA: an R package for weighted correlation network analysis. BMC Bioinform.

[16] Ohori Tatsuo G, Riu Hamada M, Gondo T, Hamada R (2009). Nomogram as predictive model in clinical practice. Gan To Kagaku Ryoho.

[17] Subramanian A, Tamayo P, Mootha VK, Mukherjee S, Ebert BL, Gillette MA, Paulovich A, Pomeroy SL, Golub TR, Lander ES, Mesirov JP (2005). Gene set enrichment analysis: a knowledge-based approach for interpreting genome-wide expression profiles. Proc Natl Acad Sci USA.

[18] Mootha VK, Lindgren CM, Eriksson KF, Subramanian A, Sihag S, Lehar J, Puigserver P, Carlsson E, Ridderstrale M, Laurila E (2003). PGC-1alpha-responsive genes involved in oxidative phosphorylation are coordinately downregulated in human diabetes. Nat Genet.

[19] Robinson MD, McCarthy DJ, Smyth GK (2010). edgeR: a Bioconductor package for differential expression analysis of digital gene expression data. Bioinformatics.

[20] McCarthy DJ, Chen Y, Smyth GK (2012). Differential expression analysis of multifactor RNA-Seq experiments with respect to biological variation. Nucleic Acids Res.

[21] Cuylen S, Blaukopf C, Politi AZ, Muller-Reichert T, Neumann B, Poser I, Ellenberg J, Hyman AA, Gerlich DW (2016). Ki-67 acts as a biological surfactant to disperse mitotic chromosomes. Nature.

[22] Yu Z, Wang R, Chen F, Wang J, Huang X (2018). Five novel oncogenic signatures could be utilized as AFP-related diagnostic biomarkers for hepatocellular carcinoma based on next-generation sequencing. Dig Dis Sci.

[23] Shi W, Hu J, Zhu S, Shen X, Zhang X, Yang C, Gao H, Zhang H (2015). Expression of MTA2 and Ki-67 in hepatocellular carcinoma and their correlation with prognosis. Int J Clin Exp Pathol.

[24] Huang C, Wang Y, Liu S, Ding G, Liu W, Zhou J, Kuang M, Ji Y, Kondo T, Fan J (2013). Quantitative proteomic analysis identified paraoxonase 1 as a novel serum biomarker for microvascular invasion in hepatocellular carcinoma. J Proteome Res.

[25] Masaki T, Shiratori Y, Rengifo W, Igarashi K, Matsumoto K, Nishioka M, Hatanaka Y, Omata M (2000). Hepatocellular carcinoma cell cycle: study of Long-Evans cinnamon rats. Hepatology.

[26] Sagawa H, Naiki-Ito A, Kato H, Naiki T, Yamashita Y, Suzuki S, Sato S, Shiomi K, Kato A, Kuno T (2015). Connexin 32 and luteolin play protective roles in non-alcoholic steatohepatitis development and its related hepatocarcinogenesis in rats. Carcinogenesis.

[27] Hine D, Mackness B, Mackness M (2012). Coincubation of PON1, APO A1, and LCAT increases the time HDL is able to prevent LDL oxidation. IUBMB Life.

[28] Schneider G, Schmidt-Supprian M, Rad R, Saur D (2017). Tissue-specific tumorigenesis: context matters. Nat Rev Cancer.

[29] Akkiz H, Kuran S, Akgollu E, Uskudar O, Bekar A, Bayram S, Yildirim S, Ulger Y, Kaya BY, Sansal M, Cinar E (2013). Effect of PON1 gene polymorphisms in Turkish patients with hepatocellular carcinoma. Meta Gene.

[30] Shu H, Li W, Shang S, Qin X, Zhang S, Liu Y (2017). Diagnosis of AFP-negative early-stage hepatocellular carcinoma using Fuc-PON1. Discov Med.

[31] Fedelesova M, Kupcova V, Luha J, Turecky L (2017). Paraoxonase activity in sera of patients with non-alcoholic fatty liver disease. Bratisl Lek Listy.

[32] Wang B, Yang RN, Zhu YR, Xing JC, Lou XW, He YJ, Ding QL, Zhang MY, Qiu H (2017). Involvement of xanthine oxidase and paraoxonase 1 in the process of oxidative stress in nonalcoholic fatty liver disease. Mol Med Rep.

[33] Baffy G, Brunt EM, Caldwell SH (2012). Hepatocellular carcinoma in non-alcoholic fatty liver disease: an emerging menace. J Hepatol.

[34] Ma C, Kesarwala AH, Eggert T, Medina-Echeverz J, Kleiner DE, Jin P, Stroncek DF, Terabe M, Kapoor V, ElGindi M (2016). NAFLD causes selective CD4(+) T lymphocyte loss and promotes hepatocarcinogenesis. Nature.

[35] Luo Y, Coskun V, Liang A, Yu J, Cheng L, Ge W, Shi Z, Zhang K, Li C, Cui Y (2015). Single-cell transcriptome analyses reveal signals to activate dormant neural stem cells. Cell.

[36] Alim Z, Kilic D, Demir Y (2018). Some indazoles reduced the activity of human serum paraoxonase 1, an antioxidant enzyme: in vitro inhibition and molecular modeling studies. Arch Physiol Biochem.

[37] Davies HG, Richter RJ, Keifer M, Broomfield CA, Sowalla J, Furlong CE (1996). The effect of the human serum paraoxonase polymorphism is reversed with diazoxon, soman and sarin. Nat Genet.

[38] Husni ME, Tang WHW, Lucke M, Chandrasekharan UM, Brennan DM, Hazen SL (2018). HDL associated paraoxonase-1 activity correlates with systemic inflammation, disease activity and cardiovascular risk factors in psoriatic disease. Arthritis Rheumatol.

[39] Garcia-Heredia A, Marsillach J, Rull A, Triguero I, Fort I, Mackness B, Mackness M, Shih DM, Joven J, Camps J (2013). Paraoxonase-1 inhibits oxidized low-density lipoprotein-induced metabolic alterations and apoptosis in endothelial cells: a nondirected metabolomic study. Mediat Inflamm.

[40] Metzker ML (2010). Sequencing technologies—the next generation. Nat Rev Genet.

[41] Hardwick SA, Deveson IW, Mercer TR (2017). Reference standards for next-generation sequencing. Nat Rev Genet.

[42] Xu RH, Wei W, Krawczyk M, Wang W, Luo H, Flagg K, Yi S, Shi W, Quan Q, Li K (2017). Circulating tumour DNA methylation markers for diagnosis and prognosis of hepatocellular carcinoma. Nat Mater.

[43] Kamerkar S, LeBleu VS, Sugimoto H, Yang S, Ruivo CF, Melo SA, Lee JJ, Kalluri R (2017). Exosomes facilitate therapeutic targeting of oncogenic KRAS in pancreatic cancer. Nature.

[44] Hoshino A, Costa-Silva B, Shen TL, Rodrigues G, Hashimoto A, Mark MT, Molina H, Kohsaka S, Di Giannatale A, Ceder S (2015). Tumour exosome integrins determine organotropic metastasis. Nature.

[45] Abdel Wahab AHA, El-Halawany MS, Emam AA, Elfiky A, Abd Elmageed ZY (2017). Identification of circulating protein biomarkers in patients with hepatocellular carcinoma concomitantly infected with chronic hepatitis C virus. Biomarkers.

[46] Suszynska-Zajczyk J, Jakubowski H (2014). Paraoxonase 1 and dietary hyperhomocysteinemia modulate the expression of mouse proteins involved in liver homeostasis. Acta Biochim Pol.

